# Multi-omics integration for neuroblastoma clinical endpoint prediction

**DOI:** 10.1186/s13062-018-0207-8

**Published:** 2018-04-03

**Authors:** Margherita Francescatto, Marco Chierici, Setareh Rezvan Dezfooli, Alessandro Zandonà, Giuseppe Jurman, Cesare Furlanello

**Affiliations:** 1 Fondazione Bruno Kessler, Via Sommarive 18, Trento, 38123 Italy; 2 Centre for Integrative Biology, University of Trento, Via Sommarive 9, Trento, 38123 Italy; 3 Department of Information Engineering, University of Padova, Padova, Italy

**Keywords:** Neuroblastoma, Integration, Prediction, Classification, Autoencoder

## Abstract

**Background:**

High-throughput methodologies such as microarrays and next-generation sequencing are routinely used in cancer research, generating complex data at different *omics* layers. The effective integration of *omics* data could provide a broader insight into the mechanisms of cancer biology, helping researchers and clinicians to develop personalized therapies.

**Results:**

In the context of CAMDA 2017 Neuroblastoma Data Integration challenge, we explore the use of Integrative Network Fusion (INF), a bioinformatics framework combining a similarity network fusion with machine learning for the integration of multiple *omics* data. We apply the INF framework for the prediction of neuroblastoma patient outcome, integrating RNA-Seq, microarray and array comparative genomic hybridization data. We additionally explore the use of autoencoders as a method to integrate microarray expression and copy number data.

**Conclusions:**

The INF method is effective for the integration of multiple data sources providing compact feature signatures for patient classification with performances comparable to other methods. Latent space representation of the integrated data provided by the autoencoder approach gives promising results, both by improving classification on survival endpoints and by providing means to discover two groups of patients characterized by distinct overall survival (OS) curves.

**Reviewers:**

This article was reviewed by Djork-Arné Clevert and Tieliu Shi.

**Electronic supplementary material:**

The online version of this article (10.1186/s13062-018-0207-8) contains supplementary material, which is available to authorized users.

## Background

Neuroblastoma is a rare disease typically manifesting in early infancy with an estimated 700 new cases diagnosed in the U.S. each year [1]. It is characterized by a very heterogeneous clinical course, with extreme cases presenting spontaneous regression opposed by patients relapsing and eventually dying despite prompt therapy [2]. Because of this heterogeneity, the ability to accurately predict the most likely disease outcome at the time of diagnosis is of extreme importance, especially given that accurate risk estimation allows delivering an appropriate targeted therapy [3]. Amplification of the oncogene *MYCN* and age at diagnosis are currently key clinical characteristics for the patient’s risk assessment [4]. However, these indicators only cover a portion of all neuroblastoma cases (ca. 22% of all neuroblastoma tumors present *MYCN* amplification [2]).

The introduction of genome wide assays able to probe in great detail multiple genomics aspects often at affordable prices brought the promise of novel biomarker identification for clinical outcome prediction, notably in combination with effective data analysis [5, 6]. Machine learning approaches have been adopted for the predictive classification of patient outcome in neuroblastoma, also through integration of data from multiple assays [5, 7]. For example, in a previous effort, the MicroArray/Sequencing Quality Control (MAQC/SEQC) initiative extensively explored expression-based predictive models for neuroblastoma risk assessment [8]. However, comprehensive integrative approaches effective across multiple clinical outcomes are still limited [5].

In the context of the CAMDA 2017 Neuroblastoma Data Integration challenge, three types of *omics* data were made available for a cohort of 145 neuroblastoma patients: microarray and RNA-Seq expression profiling and array comparative genomic hybridization (aCGH) copy number variant (CNV) profiling. For a larger set of 498 neuroblastoma patients, expression profiling by both microarray and RNA-Seq was provided, but aCGH was not available. The clinical characteristics of the patients are provided as supplementary material. In this paper, we evaluate multiple integration approaches for neuroblastoma endpoint classification, considering in particular the INF method.

INF is a recent modeling approach for the integration of multiple data types in a machine learning setting [9], originally applied to metagenomic data. On the CAMDA 2017 Neuroblastoma dataset, INF improved prediction of Event-Free Survival (EFS) endpoint on combined microarray and aCGH data with respect to both simple juxtaposition and the use of the distinct datasets independently. For the remaining endpoints and on the full set of 498 samples, classification results were more heterogeneous, with performances displaying large variation across endpoints, as previously observed [8]. Globally, INF showed the capability of extracting top feature sets significantly more compact than those identified by other methods, with almost negligible loss of classification performance. Interestingly, for each endpoint and data subset we identified subgroups of patients consistently misclassified. We additionally explored autoencoders as a deep learning approach to the integration of microarray and aCGH data. By minimizing the mean squared error objective function, we identified a latent space representation of the juxtaposed dataset able to improve classification on ALL-EFS and ALL-OS endpoints. We additionally used this representation to define two groups of patients characterized by distinct survival curves.

## Methods

The datasets used in this study include RNA-Seq and Agilent microarray gene expression profiles of 498 neuroblastoma patients [8], as well as matched aCGH data for a subset of 145 patients [10–13]. The clinical characteristics of the 498 samples were described previously [8] and are included in Additional file 1: Table S1. The following prognostic endpoints were considered for the classification tasks: the occurrence of an event (progression, relapse or death) (ALL-EFS); the occurrence of death from disease (ALL-OS); an extreme disease outcome (CLASS); the occurrence of an event (HR-EFS) and death from disease (HR-OS) in the subset of high-risk (HR) patients. The HR status was defined according to the NB2004 risk stratification criteria. Samples were split into train (TR) and test (TS) sets according to previous partitioning [8]. Outcome stratification statistics are summarized in Table 1. The clinical characteristics of the patients are provided as Additional file 1.

**Table 1:** Sample stratification (number of subjects)

Endpoint	498 cohort		145 cohort	
	TR	TS	TR	TS
ALL-EFS	249	249	71	74
ALL-OS				
CLASS	136	136	42	45
HR-EFS	86	90	NA	NA
HR-OS				

### Data processing

The RNA-Seq data was downloaded from CAMDA2017 website (http://camda2017.bioinf.jku.at/doku.php). The data provided was already preprocessed, normalized and *log*_2_ transformed using the Magic-AceView (“MAV”) pipeline, described in detail in [8]. In particular, we used the data aggregated at the gene level (“MAV-G”). Agilent microarray raw data was background-corrected (“normexp” method) and quantile-normalized with the *limma* R/Bioconductor package [14] to obtain *log*_2_ expressions for probes, further summarized over genes (“AG1-G”) using the microarray annotation file. The aCGH raw data was downloaded from GEO (accession numbers GSE45480, GSE56109, GSE25771 and GSE35953) and the file provided in Additional file 2: Table S2 was used to select and match the samples for which also microarray and RNA-Seq data was available. The selected aCGH microarray raw data files were preprocessed independently using the *rCGH* R/Bioconductor package [15] with default parameters, and segmentation tables were then summarized over genes (“CNV-G”). Features with undefined values (NA) were removed from all datasets before proceeding with downstream analyses. In addition, all data tables were filtered removing features with zero or near-zero variance using the *nearZeroVar* function in the *caret* R package with default parameters. To avoid information leakage, feature filtering was performed on the TR data set and applied on both TR and TS data sets. For the integrative analysis, juxtaposed (*juxt*) datasets AG1-G/CNV-G, AG1-G/MAV-G and CNV-G/MAV-G were created concatenating AG1-G and CNV-G, AG1-G and MAV-G, and CNV-G and MAV-G respectively.

### Predictive classification

To ensure reproducibility and control overfitting, we adopted a Data Analysis Protocol (DAP) following the guidelines proposed by the U.S. FDA-led MAQC/SEQC initiatives [16, 17] for reproducibility in the analysis of high-throughput data. Briefly, given a dataset split in TR and TS portions, the former undergoes a 10×5−fold stratified Cross-Validation (CV) resulting in a ranked feature list and an average classification performance measure, here the Matthews Correlation Coefficient (MCC) [18, 19]. As classifiers, we used Linear Support Vector Machines (LSVM) and Random Forest (RF). At each CV iteration, features were ranked by support vector machine (SVM) weights or RF Gini index and the classifier was trained on an increasing number of ranked features (in this case, [ 5,10,25,50,75,100,500,1000,5000,10000,NF], with NF indicating the total number of features in the dataset). Features were also ranked using ANOVA F-Score (“KBest” in the following) as an alternative method independent of the classifier. The ranked CV lists were then aggregated into a single ranked feature list using the Borda method [20, 21]. The best model was later retrained on the whole TR set restricted to the features yielding the maximum MCC in CV, and selected for validation on the TS set. As a sanity check to avoid unwanted selection bias effects, the DAP was repeated stochastically scrambling the TR labels (“random label” scheme). We use MCC _val_ to indicate MCC in validation, while MCC _internal_ or MCC _CV_ are used interchangeably to indicate performance in CV.

### Integrative network fusion – INF

**Fig. 1:**
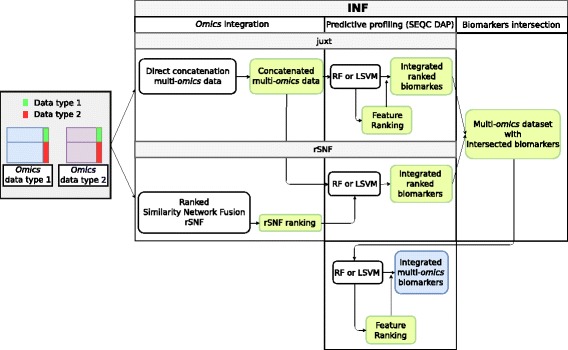
INF workflow. Graphical representation of the INF workflow for two generic *omics* datasets (adapted from [9]). A first RF classifier is trained on the juxtaposed data and the feature list obtained is ranked by mean decrease in Gini impurity (ML-*juxt*). The two data sets are then integrated by Similarity Network Fusion, the features are ranked by *rSNF* and a RF model is developed on the juxtaposed dataset with the feature ranking so defined (ML-*rSNF*). Finally, a RF classifier is trained on the juxtaposed dataset restricted to the intersection of *juxt* and *rSNF* top discriminant feature lists. All the predictive models are developed within the DAP described in the methods

We consider INF, a bioinformatics framework for the identification of integrated multi-*omics* biomarkers based on predictive profiling and a novel approach to their integration [9] (Fig. 1). In summary, first a RF (resp. LSVM) classifier is trained on the dataset obtained by juxtaposition of two *omics* data types (*juxt*), obtaining a feature list ranked by either mean decrease in Gini impurity (resp. SVM weights), or ANOVA F-Score. Secondly, the two *omics* data sets are integrated by Similarity Network Fusion [22] and features are ranked by a novel ranking scheme (*rSNF*) based on SNF-fused network clustering; a RF (resp. LSVM) model is then developed on the juxtaposed dataset with *rSNF* as feature ranking. From both approaches, a subset of top discriminant features is identified, according to the predictive performance of the classifiers. Finally, a RF (resp. LSVM) classifier is trained on the juxtaposed dataset restricted to the intersection of *juxt* and *rSNF* feature lists (*INF*). Predictive models are developed inside the DAP described above. The code implementing INF is available as a GitHub repository https://github.com/AleZandona/INF (manuscript in preparation).

### Integration evaluation

**ΔMCC** Given that classification performance across endpoints varies greatly, to evaluate multiple integration approaches we introduce the concept of *Δ*MCC, i.e. the difference between the maximum MCC on the integrated dataset and the maximum MCC on the single (non integrated) datasets. For each classifier, endpoint, and subset, given two *omics* layers *O*_1_ and *O*_2_ we define MCC on single and integrated datasets respectively as:

$$\begin{aligned} \text{MCC}_{\text{single}}&=\!\max\!\left(\text{MCC}\left(O_{1}\right), \text{MCC}\left(O_{2}\right)\right)\\ \text{MCC}_{\text{integration}}&=\!\max\!\left(\text{MCC}_{\text{juxt}}\!\left(\!O_{1},\!O_{2}\!\right)\!, \text{MCC}_{\text{rSNF}}\!\left(\!O_{1}\!,\!O_{2}\right),\right.\\ &\left.\quad \text{MCC}_{\text{INF}}\left(O_{1},O_{2}\right)\right) \end{aligned} $$

where MCC (*O*_*i*_) indicates the MCC on the single *O*_*i*_*omics* layer, and MCC_approach_(*O*_*i*_,*O*_*j*_) the MCC on the two *omics* layers *O*_*i*_,*O*_*j*_ integrated by approach ={juxt,rSNF,INF}. To evaluate the general impact of integration on classification performance, independently on the method used, we define *Δ*MCC as:

$$\begin{array}{*{20}l} \Delta \text{MCC} &= \text{MCC}_{\text{integration}} - \text{MCC}_{\text{single}} \end{array} $$

We note that the same definition was used for MCC in CV and validation.

**Mixedness** We introduce the concept of feature “mixedness” to quantify the contribution of each *omics* layer to the integrated feature set. We define the mixedness as Prop50 = percentage (%) of the layer contributing less features to the integration. With this definition, percentages closer to 50 indicate that the top feature sets are equilibrated, i.e. they acquire information from both layers. Percentages close to 0 indicate that most of the information is acquired from one of the two layers being integrated.

**Performance similarity between integration approaches** In this manuscript we compare INF performance with respect to either *juxt* or *rSNF* integration approaches. We distinguish two scenarios (we indicate with “Nfeat” the number of top features identified):

1. MCC _internal_ (or MCC _val_ or Nfeat) is equal between INF and *juxt* or *rSNF*;
2. MCC _INF_− MCC _*juxt*_<0.1 or MCC _INF_−MCC_*rSNF*_<0.1

This convention was used as color code for Additional file 3: Tables S3 and S4, with green background indicating scenario 1, and yellow scenario 2.

### Integration by deep learning

As alternative multi-*omics* integration approach, we explored the use of a deep learning autoencoder architecture inspired by the work of Chaudhary and colleagues [23]. We focused on the *juxt* AG1-G/CNV-G dataset, preprocessed as described above. We tested different autoencoder layouts, with either one, two or three fully connected layers and bottleneck sizes of 391 (one- and two-layer autoencoders) or 64 (three-layer autoencoder). For each, we experimented multiple combinations of activation functions (working with tanh, softsign and relu), two data scaling variants (minmax in either (0,1) or (-1,1)) and the introduction of L1 activation regularization terms with a range of penalties (C = 10e-6, 10e-5, 10e-4, 10e-3, 10e-2, 10e-1). For all the architectures we used the ADADELTA [24] optimizer, the mean squared error objective function and a batch size of 71. All models were trained for 2500 epochs on the TR AG1-G/CNV-G *juxt* dataset. The goodness of reconstruction was evaluated on the juxtaposed TS dataset by computing the cosine distance between reconstructed and original data (0 indicating perfect reconstruction).

**Cox regression and classification** The encoded representations of TR and TS data for the autoencoder optimizing the loss function were used for LSVM classification of ALL-EFS and ALL-OS endpoints. In addition, the meta-features of the encoded representation of the input TR data were used to fit a univariate Cox Proportional-Hazards (Cox-PH) regression model for patients’ OS. An encoded representation of the TS data was obtained from the bottleneck layer of the autoencoder fitted on the TR data. K-means clustering was applied independently to the TR and TS set meta-features significantly associated with OS to separate the TR and TS samples into two groups (the optimal number of clusters was identified using the Silhouette index (*fpc* R package) applied independently on TR and TS meta-features). Using the new sample labels so identified as target variable, an LSVM classifier was trained on the juxtaposed AG1-G/CNV-G dataset.

### Computational details

The DAP is written in Python/Scikit-Learn [25]. The autoencoder network is implemented in Keras (v. 2.1.3) [26]. Cox regression and survival analyses were performed in the R statistical environment (v. 3.3.3) [27] using the *survival* and *survminer* libraries. Plots were produced using the *ggplot2* R package. The DAP and INF were run on a 32-core Intel Xeon Linux workstation. DL computations were run on a Microsoft Azure platform with 2x NVIDIA Tesla K80 GPUs.

## Results

### Classification on the single datasets

**Fig. 2:**
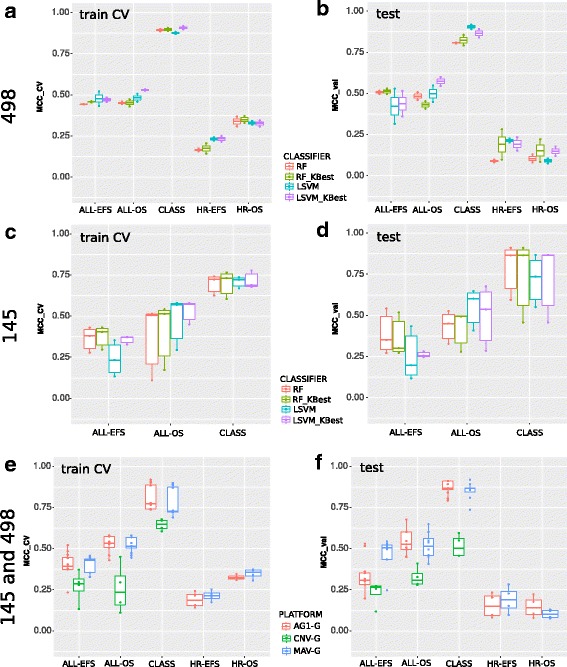
Classification performance on all endpoints considered in the study: by classifier for the 498 (panels **a** and **b**) and the 145 (panels **c** and **d**) sample subsets, as well as by platform (panels **e** and **f**) for both sample subsets

We first applied RF and LSVM classifiers, with both native and KBest feature ranking (see Methods), to the 498 and 145 datasets independently. As labels the endpoints originally proposed in [8] and summarized in Table 1 were used. In general, both classifiers achieved similar performances, independently of the ranking scheme. Consistently with previously published results [8], both classifiers achieved poor MCC performance on HR endpoints (Fig. 2, panels a and b). The best results were obtained for the CLASS label, identifying patients with extremely positive or negative disease outcomes (Fig. 2). Analogous results were obtained for the subset of 145 patients for which also aCGH data was available, with CLASS being the best performing endpoint (Fig. 2, panels c and d). Classification in this subset of the data had generally lower performance, likely due to the reduced number of samples available. We note that for this subset of the data we did not consider the HR-OS and HR-EFS endpoints, as the number of samples is too low to allow accurate prediction. Predictions based on CNV data alone were generally poor while AG1 and MAV performed better and comparably between them (Fig. 2, panels e and f).

### Integration of multiple data sources marginally improves endpoint prediction

**Fig. 3:**
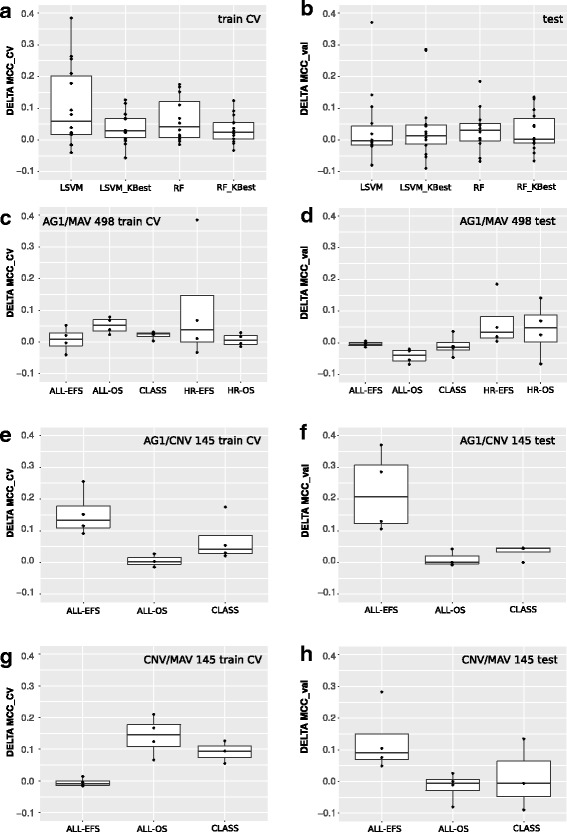
Integration evaluation. Distribution of *Δ*MCC values in cross-validation (panels **a**, **c**, **e**, **g**) and in validation (panels **b**, **d**, **f**, **h**) stratified by classifier (**a**, **b**) and endpoint (**c**–**h**). Panels **c**, **d**: AG1-G/MAV-G on the 498 data subset. Panels **e**, **f**: AG1-G/CNV-G. Panels **g**, **h**: MAV-G/CNV-G

To evaluate the overall effect of data integration with respect to classification using the single datasets independently, we introduced the concept of *Δ*MCC (see Methods). *Δ*MCC measures the difference between MCC for classification in single datasets as opposed to integrated datasets, without considering the specific method used for the integration. As shown in Fig. 3 (panels a and b) the behavior is not homogeneous: in some cases MCC improved with integration (*Δ*MCC>0) but it decreased in others. The choice of classifier does not seem to affect this behavior. Ascertained this, we present further results separated by endpoint, since we previously observed marked differences in classification performance for different endpoints. We also expected that the data types being integrated should differently affect the performance and thus we consider separately different integration settings. Since AG1-G and MAV-G essentially represent two types of measurement for the same quantity (both assays measure expression and, in this application, both of them are summarized at the gene level), we were not surprised in finding *Δ*MCC≃0 for their integration (Fig. 3, panels c and d). The most interesting integration cases are those mixing expression with CNVs, as they represent distinct *omics* layers. Integrating AG1-G and CNV-G data clearly improved the classification performance for ALL-EFS endpoint but did not impact ALL-OS and CLASS. Remarkably, performances in CNV-G/MAV-G integration did not show the same trend (Fig. 3 panels e to h).

### INF performs similarly to *juxt* and *rSNF*, but produces compact feature sets

**Fig. 4:**
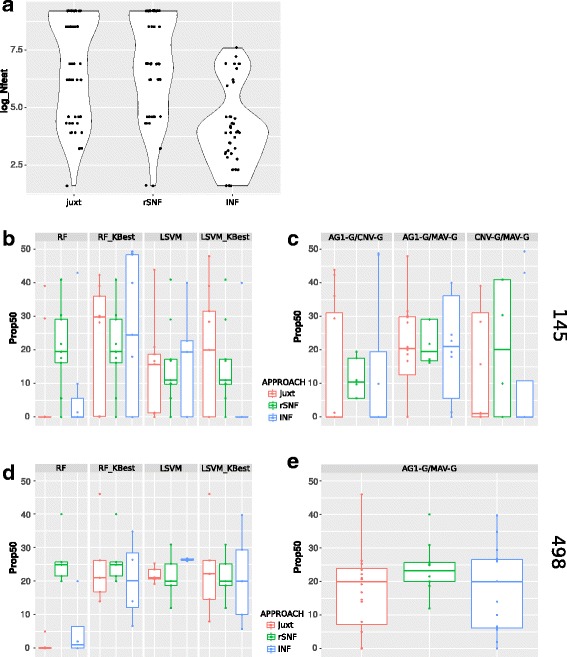
Integrated feature set sizes and mixedness. **a**. Feature set sizes by integration approach for all models. The feature sets identified by INF are more compact than those identified by *juxt* and *rSNF* (*p*-values = 2.453e-08 and 3.803e-09 respectively, Wilcoxon rank sum test). **b**. and **c**. Mixedness for the 145 data subset. **d**. and **e**. Mixedness for the 498 data subset

We compared the INF classification performance and feature sets identified with respect to simple juxtaposition (*juxt*) and *rSNF* across all subsets, endpoints and classifiers (Additional file 4). As shown in Fig. 4, the feature sets identified by INF were generally more compact than those extracted by either *juxt* or *rSNF* (*p*-values = 2.453e-08 and 3.803e-09 respectively, Wilcoxon rank sum test). The specific results for all classifiers, methods and subsets are available in Additional file 4. We note that 15 INF runs failed, either because the intersection of top features was empty or too small to be considered for classification (<5). This leaves a total of 41 runs that can be used to compare performance (as MCC either in CV or in validation). In CV, INF performed better than *juxt* (resp. rSNF) in 33 (resp. 35) cases, i.e. in 81% (85%) of the comparisons, while it performed similarly in 7 (resp 5) cases. On external validation, INF performed better than *juxt* (*rSNF*) in 14 (16) cases out of 41, corresponding to 34% (resp. 39%) of the comparisons. Therefore, as previously found for a meta-*omics* application in [9], the major advantage of INF over simple juxtaposition and *rSNF* is a more compact feature signature at similar MCC scores.

#### Mixedness

In order to evaluate how much each layer contributes to the feature signatures identified, we introduced the concept of “mixedness” (see Methods). As shown in Fig. 4b and c, considering the 145 subset of the data, Prop50 has high variability and quasi-equal contribution from both layers is rare (Prop50≥40 in 10% of the 145 top feature sets). This behavior is independent of endpoint (not shown). The top feature sets are more equilibrated for the 498 subset (excluding RF, Fig. 4d) but quasi-equal contribution from both layers is still rare (Prop50≥40 in 6% of the 498 top feature sets). Regardless of the classifier type, we observe tighter mixedness distribution for rSNF (Fig. 4e), although with larger feature lists. In general, for AG1-G/MAV-G integrations the major contributor was always MAV-G, independently of classifier or data subset (145 or 498). For the 145 subset, in which the CNV data was available besides expression, we observed higher variation: for AG1-G/CNV-G and CNV-G/MAV-G integrations, respectively in 15 and in 9 out of 33 experiments CNV was the major contributor. We note that the integration method seems to have an impact on which data type contributes more, since the majority of top feature sets in which CNV contributes greatly (>50*%*) are derived with rSNF method (20 out of 24 top feature sets).

#### Cases in which INF has superior accuracy

Considering together the two similarity scenarios introduced in Methods (i.e. both yellow and green cells in Additional file 3: Tables S3 and S4), INF performed similarly or better than both *juxt* and *rSNF* in 7 cases for RF, in 10 cases for RF KBest, 5 cases for LSVM and 7 cases for LSVM KBest (black font in Additional file 4). Considering only similarity scenario 1 (i.e. only green cells in Additional file 3: Tables S3 and S4), INF performed better than both *juxt* and *rSNF* in:

- one case for RF (498 ALL-OS AG1-G/MAV-G)
- 3 cases for RF KBest (145 ALL-OS CNV-G/MAV-G, 498 ALL-EFS AG1-G/MAV-G, 498 ALL-OS AG1-G/MAV-G)
- one case for LSVM (145 ALL-OS AG1-G/MAV-G)
- 2 cases for LSVM KBest (145 ALL-OS AG1-G/CNV-G, 145 ALL-OS CNV-G/MAV-G).

These cases are highlighted with the bold font in Additional file 4. For AG1-G/CNV-G integration on the ALL-OS endpoint, we observe that INF, coupled with LSVM and KBest, achieves MCC _val_=0.67 for 20 features. This improves the MCC _val_=0.61 obtained by LSVM with 200 MAV-G features, the best-performing model on ALL-OS developed within the SEQC initiative [8].

### Misclassified patients

We notice that for each endpoint a subset of patients is consistently classified by all classifiers, independently on data type or integration used. Based on this observation, we extracted samples that are consistently correctly or incorrectly classified (Table 2).

**Table 2:** Number of misclassified or correctly classified patients for each data subset, endpoint and classifier

		RF		RF Kbest		LSVM		LSVM Kbest	
		CC	MC	CC	MC	CC	MC	CC	MC
145	ALL-EFS	26	12	27	14	20	16	14	14
	ALL-OS	41	12	46	12	33	8	37	8
	CLASS	30	0	30	0	26	2	26	1
498	ALL-EFS	175	67	178	61	144	105	136	79
	ALL-OS	196	47	191	47	173	76	178	61
	CLASS	119	12	121	13	123	12	122	12
	HR-EFS	27	63	41	49	33	37	28	38
	HR-OS	34	48	37	46	38	52	31	48

### A deep learning approach to *omics* integration

**Fig. 5:**
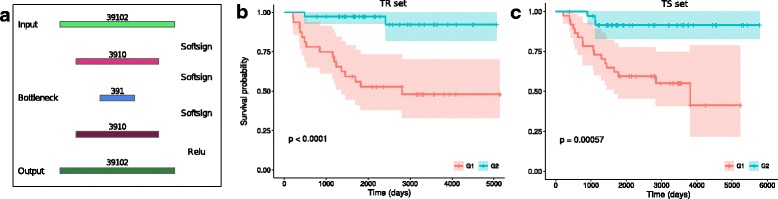
Autoencoder schematic and survival analysis. **a** Scheme of the autoencoder architecture giving the best results. **b** Kaplan-Meier survival curves for the two groups of patients identified clustering TR set autoencoder meta-features significantly associated with survival. **c** Kaplan-Meier survival curves for the two groups of patients identified clustering TS set autoencoder meta-features significantly associated with survival. The significant *p*-values suggest that the groups identify two distinct patient populations. Curves for TR/TS patients were calculated separately to highlight that the selection of survival-associated meta-feature in the TR set effectively stratifies also TS set patients

Among the architectures tested (see Methods) the best results were obtained for the two-layer autoencoder with scaling minMax(0,1), without regularization and with activation functions softsing, softsign, softsign, relu (Fig. 5a). Autoencoding of the TS set reproduced reliably the input data, as supported by cosine distance equal to 0.13. Notably, a LSVM classifier for ALL-EFS and ALL-OS endpoints trained and tested on the encoding of the juxtaposed AG1-G/CNV-G data gave better classification performance with respect to using the full dataset (Table 3). Cox-PH regression analysis on the 391 units of the bottleneck layer found 87 deep features significantly associated with OS (FDR-adjusted log-rank *p*<0.05). Out of these, 83.8% were also significantly associated with OS in the encoded representation of the TS data obtained from the bottleneck layer of the autoencoder fitted on the TR data. K-means clustering, applied independently on the TR and TS set meta-features significantly associated with OS, identified 2 optimal clusters, representing two groups of patients G1 (76 patients: 39 TR, 37 TS) and G2 (69 patients: 32 TR, 37 TS). The patient assignment to the two groups is provided in Additional file 5. As shown in Fig. 5 (b and c) the two distinct groups are characterized by significantly different survival curves. A LSVM classifier trained on the juxtaposed AG1-G/CNV-G dataset using the labels G1 and G2 defined by the clustering as target achieved MCC _val_=0.716 (MCC _CV_=0.817(0.781−0.856), Nfeat = 1000).

**Table 3:** Comparison of classification performance on ALL-EFS and ALL-OS endpoints using the *juxt* AG1-G/CNV-G dataset or its embedding obtained using the autoencoder approach

	AG1-G/CNV-G			AG1-G/CNV-G encoding		
Endpoint	MCC _CV_	MCC _val_	Nfeat	MCC _CV_	MCC _val_	Nfeat
ALL-EFS	0.207 (0.141-0.278)	0.197	1000	0.438 (0.374-0.503)	0.360	100
ALL-OS	0.523 (0.443-0.591)	0.359	500	0.545 (0.466- 0.618)	0.538	100

## Discussion

We introduced the INF framework for multi-*omics* cancer data integration, with a first application to the neuroblastoma data made available for the CAMDA 2017 challenge. We aimed at improving technical aspects, performance and biological insights on this dataset. In general integration seems to improve inconsistently the prediction performance. We tried to integrate three data types, two of which are redundant (both MAV and AG1 provide expression measures). Although CNVs perform poorly alone as a classifier, their integration with microarray data improves classification in some cases. Interestingly, for each endpoint and data subset we identified a set of patients that are consistently misclassified, independently from integration strategy, assay, clinico-genetic subgroups and INSS staging. This opens the intriguing possibility that these patients could represent a subgroup characterized by distinctive biomarkers. The deep learning approach for prototype *omics*-integration framework identifies a new label, that distinguishes two groups of patients with distinct survival curves.

## Conclusions

As novel method for the integration of multiple *omics* data, the INF method is applied to the three datasets proposed for the CAMDA 2017 Neuroblastoma Data Integration challenge. We compared INF classification performance with simple juxtaposition and rSNF, proving that it performs comparably or better than either in most cases, with the advantage of very compact feature sets (on average 75% reduction with similar accuracy). We additionally tested an *omics*-integration framework based on deep learning to identify a novel set of “meta-features” able to distinguish patient groups with markedly different survival curves. The relationship between meta-features derived from the deep learning autoencoder and the INF features is currently under development.

## Reviewers’ comments

### Reviewer’s report 1: Djork-Arné Clevert, Bioinformatics Department, Bayer AG, Berlin, Germany

**Reviewer comment:** Quality of written English. Needs some language corrections before being published.

Author’s response: *we carefully revised the English used in the manuscript.*

**Reviewer comment:** Francescatto et al. describe in this paper the usage of Integrative Network Fusion and an unsupervised Deep Learning approach for representational learning to analyse multi-omics data in the context of CAMDA 2018’s Challenge. The challenge data set comprises partly matched aCGH, RNA-seq and microarray gene expression profiles for clinical endpoint prediction of 498 children patients. The manuscript is written in a very clear and understandable way and is methodically well prepared.

Author’s response: *We thank the reviewer for critically evaluating our work and for the positive feedback*.

**Reviewer comment:** The data preprocessing and RNA-Seq data might have been improved by variance stabilizing normalization, but overall there is nothing wrong with the pipeline used.

Author’s response: *We note that the RNA-seq data was provided by CAMDA2017 organizers, already preprocessed, normalized and log2 transformed. The approach used, originally described in Zhang et al. 2015, follows the Magic-AceView pipeline, which includes quantification and normalization of the RNA-seq data. We agree that this was not clearly explained, thus we have accordingly updated the “**Data processing**”* *Methods* *subsection in order to include additional information.*

**Reviewer comment:** Furthermore, the filter for low-variance features was only used on the training set and therefore no selection bias was introduced on the test set. Unfortunately, the section on the integration of Deep Learning is too brief and has to be described in more detail in terms of reproducibility.

Author’s response: *We thank the reviewer for pointing that the Deep Learning section was not clearly presented. We have added missing details that we understand are necessary for reproducibility. Building on the reviewer comments, we revisited the autoencoder architecture and performed additional experiments to systematically test and review alternative architectures and parameters. To validate in a reproducible way the choice of network architecture, we alternatively considered three autoencoder layouts more simple than the one proposed in the original version of the article: a single fully-connected neural layer as encoder and as decoder (AE1) and a two- (AE2) and three-layer (AE3) fully-connected autoencoders. We also experimented with the size of the bottleneck layer, as its original size (64 nodes) was possibly too small to properly capture the dataset characteristics. In particular we settled for a bottleneck of 391 nodes (1% of the number of features in input) for autoencoders AE1 and AE2, while maintaining a 64-nodes bottleneck layer for AE3. Within these architectures, we tested multiple combinations of activation functions (working with tanh, softsign and relu activations), an alternative data scaling (minMax(-1,1) in addition to the minMax(0,1) originally used) and the introduction of an L1 activity regularization term with a range of penalties* (*C*=10*e*−6,10*e*−5,10*e*−4,10*e*−3,10*e*−2,10*e*−1).*In all the experiments, we used the mean squared error as objective function and the models were trained for 2500 epochs. For each parameter combination we calculated the cosine distance between the input and its reconstruction to evaluate the goodness of the reconstruction. In terms of loss and cosine distance, the best results were obtained for autoencoder AE2 with scaling minMax(0,1), without regularization and with activation functions softsign, softsign, softsign, relu.*

**Reviewer comment:** Which learning rate scheduler was used?

Author’s response: *We used the ADADELTA optimizer, which is an adaptive learning rate method that doesn’t require manual tuning of learning rates. We updated the “Integration by Deep Learning” Methods subsection in order to include this information.*

**Reviewer comment:** How was the network regularized- was input-noise taken into consideration?

Author’s response: *Building on the comments by the reviewer, we tested the introduction in our architecture of L1 network regularization terms for penalties* *C*=0, 10*e*−6, 10*e*−5, 10*e*−4, 10*e*−3, 10*e*−2 and 10*e*−1. *We note that introducing regularization penalties* >=10*e*−6 *generally destabilized the model. Input noise was not taken into account in these experiments.*

**Reviewer comment:** Which activation functions and batch size were used?

Author’s response: *We expanded the Methods subsection “Integration by Deep Learning” in order to include further details about the architectures, including information about activation functions and batch size. We also added a schematic diagram describing the best performing architecture selected after testing multiple combinations (Fig. *5a). *As shown, we used softsign activation in all layers except the last, in which we used relu instead. Since the hardware used to run the experiments allowed us to do so, we used a batch size of 71, which allowed us to process all samples in a single batch.*

**Reviewer comment:** Furthermore, it is not clear how and at which layer the different data sources flow into the network and neither how were missing values handled during training?

Author’s response: *The two distinct data sources (microarray and aCGH data) used in the autoencoder experiments were juxtaposed and used as input layer. This information has been added to the Methods subsection “Integration by Deep Learning”. Missing values for all the datasets used in this study were removed during data preprocessing. This information, originally missing, has been added to the “Data Processing” subsection of methods.*

**Reviewer comment:** Why was the learned 64-dim representation not examined in depth? Here, the authors could have propagated the maximum for each unit back into the input layer and, for example to generate biologically insights, could have carried out a gene set enrichment analysis.

Author’s response: *This could be done, but the (biological) meaningfulness of the results would still be questionable, since the backtracking of the resulting metagenes would lead to a weighted linear combination of all genes; then, any method adopted to select the top-genes would rely on the resulting weights, which can hardly be reliably linked to a score of biological importance.*

### Reviewer’s report 2: Tieliu Shi, East China Normal University, Shanghai, China

**Reviewer comment:** 1. It seems that the INF method proposed by the authors only improved the performance for ALL-EFS, but has no obvious impact on other clinical endpoints. please explain it.

Author’s response: *We agree with the reviewer that INF does not obviously improve the classification performance for all the clinical endpoints included in this study: however, this is not the message we want to convey by our manuscript. In fact, classification performance is just one of two aspects of novelty discussed in this manuscript. The major impact (and possibly advantage) of INF lies in its capability of extracting top feature sets that are more compact than those identified by juxt and rSNF, with almost negligible loss of classification performance. This advantage is indeed critical in studies aimed at identifying small sets of biomarkers, as is often the case in studies of clinical relevance*

**Reviewer comment:** 2. In Fig. 4a, the authors concluded that the feature sets identified by INF were more compact than those identified by juxt and rSNF, suggest to conduct statistical tests to further clarify the significance level.

Author’s response: *Following the suggestion of the reviewer, we used Wilcoxon rank sum test to test the significance of the difference between the number of top features identified by INF and juxt/rSNF. We added this information to the manuscript (all differences are significant).*

**Reviewer comment:** 3. As shown in Fig. 4b-e, the mixedness is variable and rarely equilibrated, which layer made the major contribution to the integration approach? Please clarify.

Author’s response: *As the reviewer points out, it is true that mixedness is rarely equilibrated. Considering which data type contributes the most to the top features identified with the different methods, some patterns can be observed when stratifying the results in terms of data types being integrated. In particular, we note that for AG1-G/MAV-G integrations, the major contributor is always MAV-G, independently on classifier or data subset (145 or 498). For the 145 subset, in which the CNV data is available besides expression, we observe more variety: for AG1-G/CNV-G and CNV-G/MAV-G integrations, respectively in 15 and in 9 out of 33 experiments CNV is the major contributor. We note that the integration method seems to have a crucial role here, since the majority of top feature sets in which CNV contributes importantly are derived with rSNF (20 out of 24 top feature sets). We expanded the “Mixedness” Results subsection in order to clarify the composition of the top feature sets.*

## Additional files

Additional file 1 **Table S1**. Clinical characteristics of the patients included in the study. (XLSX 27.9 kb)

Additional file 2 **Table S2**. Information relative to the match of aCGH samples and RNA-Seq/microarray samples included in the study. (XLSX 9.23 kb)

Additional file 3 **Tables S3 and S4.** INF performances (MCC _CV_, MCC _val_, Nfeat) color coded to highlight cases in which INF performs better than *juxt* or *rSNF* integration approaches (sheet S3 :*juxt* vs. INF; sheet S4: *rSNF* vs. INF). Green cell background: MCC _CV_ (or MCC _val_ or Nfeat) is equal between INF and *juxt* or *rSNF*; yellow cell background: MCC _INF_−MCC_*juxt*_<0.1 or MCC _INF_−MCC_*rSNF*_<0.1; NP: not performed; NA: not available (see main text); SF: the top feature set is too small for reliable classification. (XLSX 10.5 kb)

Additional file 4 **Tables S5–S8.** The tables highlight the cases in which INF performs similarly or better than both *juxt* and *rSNF*. Each sheet represents one classifier (sheet S5: RF; sheet S6: RF KBest; sheet S7: LSVM; sheet S8: LSVM KBest). The information is coded as follows. Bold: INF performs better than both *juxt* and *rSNF* in terms of MCC _CV_, MCC _val_ and Nfeat. Black (not bold): MCC _INF_ − MCC_*juxt*_ < 0.1 and MCC _INF_ − MCC_*rSNF*_<0.1. Light gray: failed run or INF performs worse in either CV or validation. (XLSX 15.7 kb)

Additional file 5 **Table S9**. The table provides the assignment of the 145 subset patients into the two groups G1 and G2 identified with the deep learning approach and characterized by distinct survival curves. For clarity we report also the TR/TS assignment. (XLSX 7.46 kb)

## Abbreviations

aCGH: Array comparative genomic hybridization
CNV: Copy number variant
CV: Cross validation
Cox-PH: Cox proportional-hazards
DAP: Data analysis protocol
EFS: Event free survival
HR: High-risk
INF: Integrative network fusion
MAQC/SEQC: MicroArray/sequencing quality control
MAV: Magic-AceView
MCC: Matthew’
s correlation coefficient; OS: Overall survival
LSVM: Linear support vector machine
RF: Random forest
SVM: Support vector machine
TR: Train
TS: Test
